# High performing solution-coated electrolyte-gated organic field-effect transistors for aqueous media operation

**DOI:** 10.1038/srep39623

**Published:** 2016-12-22

**Authors:** Qiaoming Zhang, Francesca Leonardi, Stefano Casalini, Inés Temiño, Marta Mas-Torrent

**Affiliations:** 1 Institut de Ciència de Materials de Barcelona (ICMAB-CSIC) and CIBER-BBN, Campus de la UAB, Bellaterra, 08193, Spain; 2 School of Physical Science and Technology, Southwest University, Chongqing, 400715, People’s Republic of China

## Abstract

Since the first demonstration, the electrolyte-gated organic field-effect transistors (EGOFETs) have immediately gained much attention for the development of cutting-edge technology and they are expected to have a strong impact in the field of (bio-)sensors. However EGOFETs directly expose their active material towards the aqueous media, hence a limited library of organic semiconductors is actually suitable. By using two mostly unexplored strategies in EGOFETs such as blended materials together with a printing technique, we have successfully widened this library. Our benchmarks were 6,13-bis(triisopropylsilylethynyl)pentacene and 2,8-difluoro-5,11-bis(triethylsilylethynyl)anthradithiophene (diF-TES-ADT), which have been firstly blended with polystyrene and secondly deposited by means of the bar-assisted meniscus shearing (BAMS) technique. Our approach yielded thin films (*i.e.* no thicker than 30 nm) suitable for organic electronics and stable in liquid environment. Up to date, these EGOFETs show unprecedented performances. Furthermore, an extremely harsh environment, like NaCl 1M, has been used in order to test the limit of operability of these electronic devices. Albeit an electrical worsening is observed, our devices can operate under different electrical stresses within the time frame of hours up to a week. In conclusion, our approach turns out to be a powerful tool for the EGOFET manufacturing.

Aiming at the fabrication of low-power electronics (*i.e.* <1 V), the electrolyte-gated organic field effect transistors (EGOFETs) represent one of the latest breakthroughs, whose layout consists of exposing directly the organic semiconductor (OSC) towards aqueous media without the need of an encapsulation layer^1^. The operational mechanism of these devices shares the same features widely studied in ions sensitive field-effect transistors^2^,^3^. In particular, the two electrical double layers (EDLs) established at both gate electrode/water and water/OSC interfaces are responsible for the electrical tuning of EGOFETs^1^,^4^. Since the aqueous media acts as the effective gate dielectric, a drastic lowering of the operational voltages is intrinsically guaranteed, because the usual EDL capacitance spans from few up to hundreds of μF/cm^2^ compared to nF/cm^2^ of standard dielectrics^5^. One direct consequence was the extensive exploitation of surface treatments on both gate electrode and OSC, which directly affects the overall performance. Due to such interface sensitivity and water compatibility, EGOFETs can be turned into (bio)-transducers^4^,^6^. Accordingly, it has been possible to monitor different biological events such as host-guest recognition^7^,^8^, DNA hybridization^9^, or electro-catalysis of metabolites^10^, whose natural environment is the aqueous media. It was even demonstrated the potential to release drugs by playing with the electrical fields governing these types of devices^11^. Furthermore, EGOFETs belong to the family of the organic transistors, which means that these (bio)-transducers take advantage of their technical pros. Among them, organic field-effect transistors provide a multi-parametric response showing features like (i) threshold voltage (*V*_*th*_), (ii) mobility (*μ*), (iii) on/off ratio (*I*_*on*_/*I*_*off*_), (iv) subthreshold slope (*SS*), and (v) transconductance (*g*_*m*_). Although the choice of using EGOFETs has many advantages for the development of multi-functional platforms interesting in the field of diagnostics^12^ and healthcare^8^, some unsolved problems are actually hindering their technological transfer such as their electrical stability. As a consequence, it is crucial to obtain a robust and efficient device that will be the core of a technology transferable to the electronic market. To the best of our knowledge, a groundbreaking EGOFET must encompass all the following characteristics: (i) *μ* ≥ 0.1 cm^2^V^−1^s^−1^ 13 (ii) high on/off ratio ≥ 10^3^ 1 (iii) a potentiometric sensitivity in the range of μV^14^, (iv) operational frequency (*f*) higher than 200 Hz^14^ and (v) capability to withstand prolonged (*i.e.* from hours to days) electrical stresses^15^,^16^. To date, there are no precedents of EGOFETs that meet all these criteria. Undoubtedly, the choice of the OSC and its consequent processing are pivotal^4^. A high degree of crystallinity along with an extended homogeneity at long-range length scales (*i.e.* from few to hundreds of μm) is fundamental to avoid the so-called “electrochemical doping”, whose distinctive fingerprints are a marked hysteresis, electrical instability within time frame of minutes and/or hours and slow operational response^17^. With this in mind, spin-coated semi-crystalline or liquid crystalline polymer OSCs^1^,^18^ have been chosen as active layers in EGOFETs as well as single crystals^1^ or thin films^14^,^19^,^20^ of small molecule semiconductors prepared by vacuum sublimation. In principle, small molecules could potentially give rise to more highly crystalline films than polymers blocking more efficiently the diffusion of ions, but it is imperative to find low cost and scalable routes to process them. Our approach consists of combining two widely overlooked strategies in the organic electronics operated in liquid: (i) the exploitation of blends composed by an insulating polymer and a small molecule organic semiconductor^13^,^21^,^22^,^23^ and (ii) the use of a solution-shearing technique, such as bar-assisted meniscus shearing (BAMS), to deposit the OSC^24^,^25^. The former strategy is based on the exploitation of polystyrene (PS), as insulating polymer, and the benchmark soluble OSCs 6,13-bis(triisopropylsilylethynyl)pentacene and 2,8-difluoro-5,11-bis(triethylsilylethynyl)anthradithiophene (diF-TES-ADT)^26^. The use of blends of organic semiconductors with PS have been shown to promote material processability and also leads to thin films with an enhanced crystallinity and environmental stability^24^,^25^,^26^. Further, BAMS is a low cost technique compatible with roll-to-roll processes and with flexible substrates for large-area fabrication^27^. This technique has recently been demonstrated to produce high-crystalline thin films in one single step featuring electrical performances comparable to devices based on amorphous silicon^24^,^25^,^27^.

In this article, we demonstrate how our approach of combining OSC: PS blends coupled with BAMS can be successfully exploited for EGOFETs manufacturing with superior performances with respect to the state of the art. For this reason, our devices have been further challenged by using a saline solution (*viz.* NaCl 1M) in order to mimic an ionic strength comparable or even harsher than real samples. Although the EGOFETs performances are clearly affected by a more stressful media, no electrical failure is observed. This proves how our approach can be successfully exploited in the field of EGOFETs paving the way towards more complex technological platforms.

## Results and Discussion

### Electrical characterization in aqueous media

**Figure 1:**
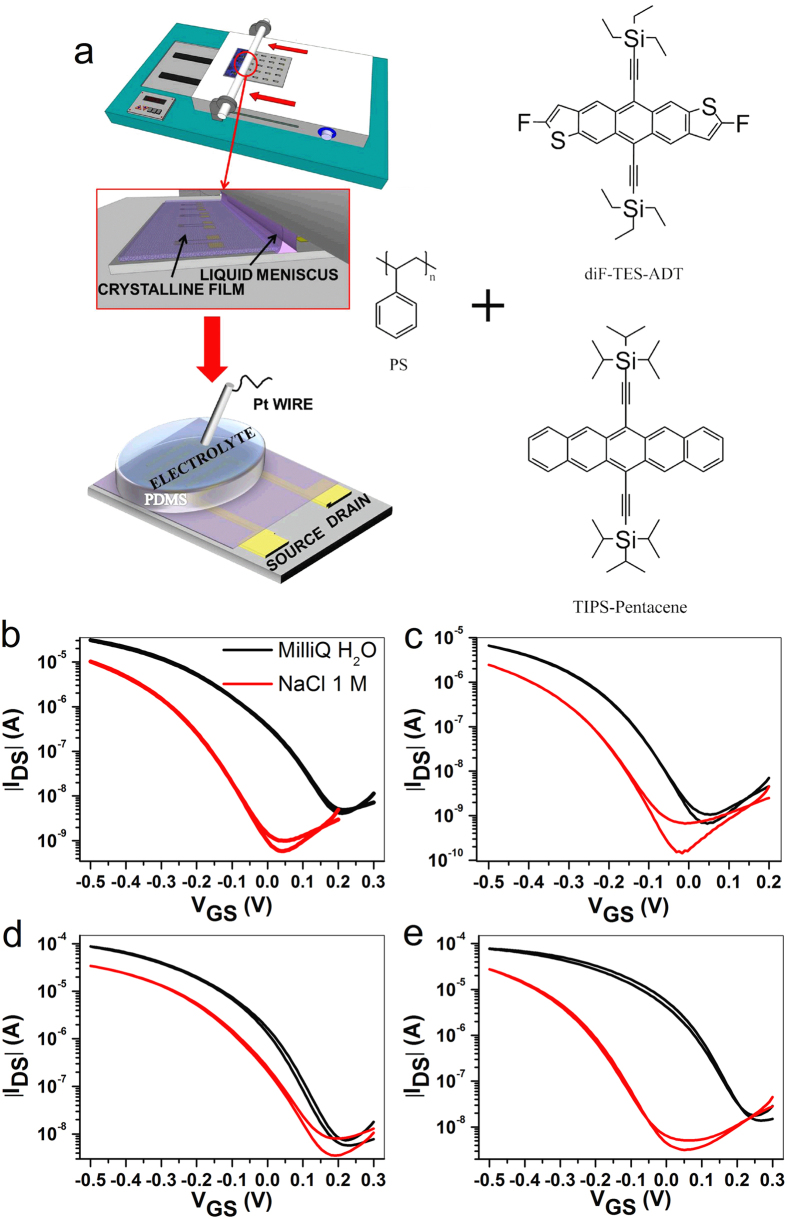
(**a**) Representative sketch of BAMS setup and EGOFET architecture along with the chemical structures of TIPS-pentacene, diF-TES-ADT and PS. *I-V* transfer characteristics of (**b**) TIPS:PS/PFBT, (**c**) TIPS:PS, (**d**) diF:PS/PFBT and (**e**) diF:PS. Electrical characteristics were recorded in saturation regime (*V*_*DS*_ = −0.4 V).

As previously mentioned, two soluble OSCs (*viz.* TIPS-pentacene and diF-TES-ADT, as shown in Fig. 1a) have been selected to be mixed with polystyrene in a 4:1 ratio in chlorobenzene (see Methods). We selected these two OSCs because they have already shown excellent performances in “standard” organic field-effect transistors^28^,^29^. Furthermore, 2,3,4,5,6-pentafluorothiophenol (PFBT) has been used as electrode modifier onto source/drain (S/D) electrodes in order to improve the electrical performances due to a better fine tuning of charge-injection and morphological homogeneity of the active material coating^30^. Our rationale consists of a systematic comparison between these two types of blends, namely TIPS-pentacene and diF-TES-ADT with polystyrene, together with PFBT-coated or uncoated S/D electrodes. For sake of clarity, we refer these 4 types of devices throughout the manuscript as follows: (i) TIPS:PS/PFBT, (ii) TIPS:PS, (iii) diF:PS/PFBT, (iv) diF:PS. These blends have been further processed by means of BAMS. This bar coating technique consists in pouring the OSC blend solution between a heated substrate and a smooth cylindrical bar positioned at a controlled distance forming a confined meniscus (Fig. 1a). Afterwards the bar is displaced along the whole extension of the substrate of interest yielding instantaneously a crystalline and uniform thin film due to the fast solvent evaporation^24^,^25^. Firstly, we checked systematically the domain size of each thin film by using the polarized optical microscope (see Fig. S1). The crystals of the semiconducting thin film do not show any dependency with respect to the bar-shearing direction because of the high deposition speed (*i.e.* 1 cm s^−1^)^25^. The crystals size shows a clear dependence with respect to the surface treatment of the S/D electrodes. As a result, both TIPS:PS/PFBT and diF:PS/PFBT domains have a size as high as 30 × 30 μm^2^; whereas in devices lacking of electrode PFBT functionalization the domains have almost double the size (~60 × 60 μm^2^), although the films are slightly less homogenous with crystallites with less regular shapes, especially in the case of TIPS (see Fig. S1). This might be due to the higher nucleation present with the more interacting PFBT-Au electrodes^30^. In order to gain information about the crystallinity of the films, X-ray powder diffraction were characterized (see Fig. S2). In TIPS:PS/PFBT and diF:PS/PFBT devices, clear sets of diffraction peaks were observed, indicative of their high degree of crystallinity. By using atomic force microscopy (AFM), we have been able to study the coating morphology in terms of thickness and root mean square roughness (*σ*_*rms*_). All the films are no thicker than 30 nm (see Fig. S3) and *σ*_*rms*_ is not affected by the presence of the two underlying materials, namely thermal SiO_x_ and polycrystalline Au (see Fig. S4). As previously mentioned, an extended homogeneity and crystallinity of the semiconducting thin film are the two desirable features for EGOFETs operability, and our coatings fulfill these basic requirements.

Moving from morphological to electrical characterization, we have tested these thin films in the EGOFET configuration, whose architecture consists of using a top electrode, namely a Pt wire immersed in the electrolytic solution, and the two Au S/D bottom contacts (Fig. 1a). The devices were electrically characterized in two types of media: (i) MilliQ water and (ii) NaCl 1M aqueous solution. The former allowed us to verify the field-effect mode of operation, whereas the latter one enables to assess their effective exploitation in real aqueous samples whose ionic strength is more complex than MilliQ water. As expected, *I-V* transfer (Fig. 1b–e) and output characteristics (Fig. S5) show a p-type behavior of these four types of devices spanning from gate voltages of 300 mV (*i.e.* OFF state) to −500 mV (*i.e.* ON state). In MilliQ water, *I-V* transfer characteristics of both OSCs do not show hysteresis and they have excellent amplification capability (namely both have *I*_*ON*_/*I*_*OFF*_ centered at or close to 10^4^, as shown in Table 1) as well as a subthreshold slope close to the theoretical threshold, that is 60 mV/decade (Table 1)^31^.

When the devices were tested in the NaCl 1M solution, no electrical failures were observed. To the best of our knowledge, this is the first time that an EGOFET shows this level of robustness. Another noticeable effect is the negative shift of the switch-on voltage (*V*_*ON*_) in NaCl 1M solution, which gives lower source-drain current (*I*_*DS*_) at a fixed gate-source voltage (*V*_*GS*_). Although the effective reason of this negative shift is beyond the scope of this work, it is known that the drastic increase of the ionic strength intrinsically triggers the so-called “ionic screening”, which has been already observed in other electronic devices operated in aqueous media^31^,^32^.

According to the classical model applied to the organic field-effect transistors, we used the following equation fulfilled in the saturation regime to calculate the devices field-effect mobility:


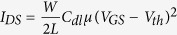


where *μ* is the charge-carriers mobility, *W* is the channel width, *L* is the channel length, *V*_*th*_ threshold voltage, *V*_*GS*_ gate-source voltage and *C*_*dl*_ capacitance of the electrical double layer.

**Figure 2:**
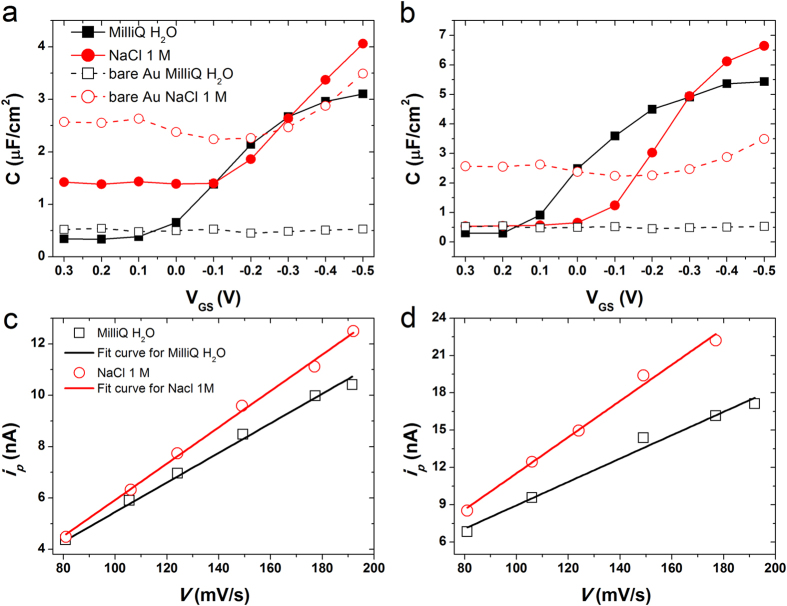
*C*_*dl*_ vs *V*_*GS*_ plots for (**a**) TIPS:PS/PFBT and (**b**) diF:PS/PFBT devices. Dashed black line (unfilled squares) is for MilliQ water and dashed red line (unfilled circles) stand for NaCl 1M related to bare Au. Solid lines (filled symbols) are the corresponding coated devices. Current peak (*i*_*p*_) versus scan rate (*v*) plots are shown for (**c**) TIPS:PS/PFBT and (**d**) diF:PS/PFBT devices. Black line and empty squares correspond to the linear fit in MilliQ water; red lines and empty circles are linear fit in NaCl 1M.

To calculate *μ*, we need first to characterize *C*_*dl*_, therefore we carried out two well-accepted tests: (i) electrochemical impedance spectroscopy (EIS)^33^, and (ii) displacement current measurement (DCM). These experiments exploit the same device architecture and the only difference resides on short-circuiting S/D electrodes. The former measurement consists of applying different set potentials scanning from 10^6^ Hz to 10^0^ Hz, whereas the latter one is based on applying the same triangular *V*_*GS*_ ramp (as used for *I-V* transfer characteristics) at different scan rates (*v*). The EIS is capable to provide *C*_*dl*_ data as a function of gate-source applied voltage and frequency (see Fig. S6a–d). *C*_*dl*_*-V*_*GS*_ graphs have been plotted by fixing a frequency equal to 10 Hz (Fig. 2a,b), as already used in similar studies^33^,^34^. As a comparison, the same experiments were performed with the bare Au without any thin film and no dependency of *C*_*dl*_ with respect to *V*_*GS*_ was observed demonstrating how the accumulation/depletion of the charge-carriers into the blended thin film is responsible for the capacitive modulation (Fig. 2a,b and Fig. S6e,f). Thus, we used a *C*_*dl*_ value of 5.3 μF/cm^2^ (6.1 μF/cm^2^) for diF:PS and 2.9 μF/cm^2^ (3.3 μF/cm^2^) for TIPS:PS in MilliQ water (and in NaCl 1M) to calculate the field-effect mobility (Fig. S7). On the other hand, as a crosscheck, DCM was employed to confirm the *C*_*dl*_extracted from EIS. The DCM experiments allowed us to monitor the peak current (*i*_*p*_) caused by the accumulation/withdrawal of the charge carriers in the OSC thin film (see Fig. S8). *i*_*p*_ vs. v plots enable to extract *C*_*dl*_ (Fig. 2c,d)^35^. The results reported in Table S1 demonstrate the good consistency of these two techniques.

Aiming at getting more information upon the PS role played in the electrical performances, other 4 devices were fabricated without PS to crosscheck, namely TIPS/PFBT, TIPS, diF/PFBT and diF. Table 1 resumes the main device characteristics for all the prepared EGOFETs. The data shown here include the average values together with the standard deviations extracted for 10 devices of each type. It is clear that the presence of both PS and PFBT are fundamental for achieving the top performances. Concerning the mobility, TIPS:PS/PFBT devices reach a *μ* equal to 0.12( ± 0.01) cm^2^V^−1^s^−1^ and diF:PS/PFBT *μ* is 0.18(±0.01) cm^2^V^−1^s^−1^ in MilliQ water. The device mobility drops between 45–60%, where the S/D electrodes have not been modified with PFBT. Furthermore, when PS is not present in the active material, the mobility dramatically decreases down to one or two orders of magnitude elucidating the major role that the polymer is playing (see Fig. S9). As previously reported, the addition of PS favors the organic semiconductor crystallization induced by the vertical phase segregation of the two materials (*i.e.*, OSC and PS) giving rise to larger crystalline domains^21^,^25^. In fact, the PS- and PFBT-free coatings show smaller and less defined crystals, respectively (see Fig. S10). The synergic combination of PFBT and PS allows us to achieve semiconducting thin films featuring an extended morphological homogeneity along with a long range crystallinity (see Fig. S1). For all these reasons, we can safely state that the best EGOFET configurations are the so-called TIPS:PS/PFBT and diF:PS/PFBT. Hence, our investigations have been further carried out only on these two types of devices.

### Potentiometric sensitivity and Switching speed

**Figure 3:**
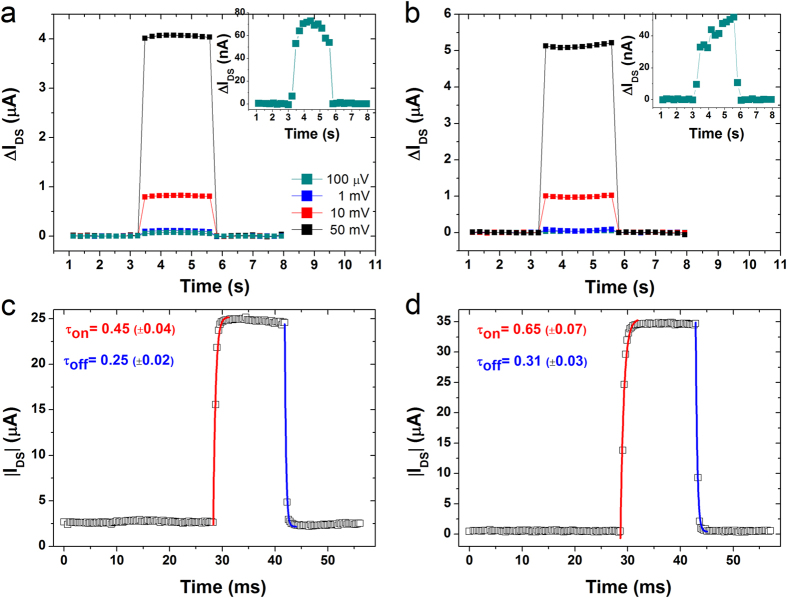
Different *I-t* plots are recorded corresponding to different step potentials (*ΔV*_*GS*_) with amplitudes equal to 50 mV, 10 mV, 1 mV and 100 μV for (**a**) TIPS:PS/PFBT and (**b**) diF:PS/PFBT devices. *I-t* plot for a step potential with amplitude equal to 0.5 V for (**c**) TIPS:PS/PFBT and (**d**) diF:PS/PFBT. All the measurements are recorded in MilliQ water at *V*_*DS*_ = −0.4 V. Red and blue lines stand for the exponential fit related to the switch on and off of the device.

Such promising results prompted us to deepen our knowledge of these devices upon figure of merits such as the potentiometric sensitivity^14^,^36^ and the dynamic response once a transient signal (namely *ΔV*_*GS*_) is applied^14^. This information plays a key role on the design of novel (bio-)transducers devoted to track some biological events, and/or can provide a precise description of what these devices can monitor in a fixed time-frame. Since MilliQ water represents our benchmark to assess the field-effect operation, we verified both TIPS:PS/PFBT and diF:PS/PFBT EGOFETs by means of different gate-source pulses (*viz. ΔV*_*GS*_) ranging from 50 mV to 100 μV as voltage amplitudes (Fig. 3a,b). The former devices turned out to be more sensitive reaching sensitivities down to 100 μV, whereas the latter ones were capable to detect *ΔV*_*GS*_ equal to 500 μV. In both cases, the signal to noise ratio was equal to 50. The increase of the ionic strength, namely moving from MilliQ water to NaCl 1M, did not affect the potentiometric sensitivity, although a worse signal to noise ratio was observed likely due to the *V*_*ON*_ shift, as previously mentioned (see Fig. S11a,b). For the switching speed (*τ*) study a voltage pulse from 0 V to −500 mV and vice versa was applied (Fig. 3c,d). The drain-source current (*I*_*DS*_) was monitored as function of time (namely *I-t* plot) throughout the experiment test. The two potential changes yield current fluctuations, which have been exponentially fitted (*i.e.* 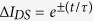) in order to quantify the effective response speed. The *τ* values are listed in Table 2. They show a slight asymmetry between switch-on (*τ*_*on*_) and switch-off (*τ*_*off*_), as already published for other EGOFETs^1^. Importantly to highlight, our devices are at least one order of magnitude faster than EGOFETs previously reported, in which *τ* was equal to 4.6 ms and 50 ms based on an evaporated film of pentacene and a rubrene single crystal, respectively^1^,^14^. All the *τ* values, here reported are lower than 1 ms, supporting the fact that no electrochemical doping is occurring and that the devices can work at frequencies higher than 1 KHz^17^. The increase of the ionic strength induces an additional switch-on acceleration, which pushes *τ* values even beyond our instrumental *I*_*DS*_ recording (*i.e.* <250 μs), as shown in Fig. S11c,d. This indicates that these systems is mainly governed by the capacitance of TIPS:PS/PFBT and diF:PS/PFBT films together with the electrolyte resistance^37^, hence the increase of the ionic strength lowers the electrolyte resistance and, consequently, *τ*.

### Stability measurements

**Figure 4:**
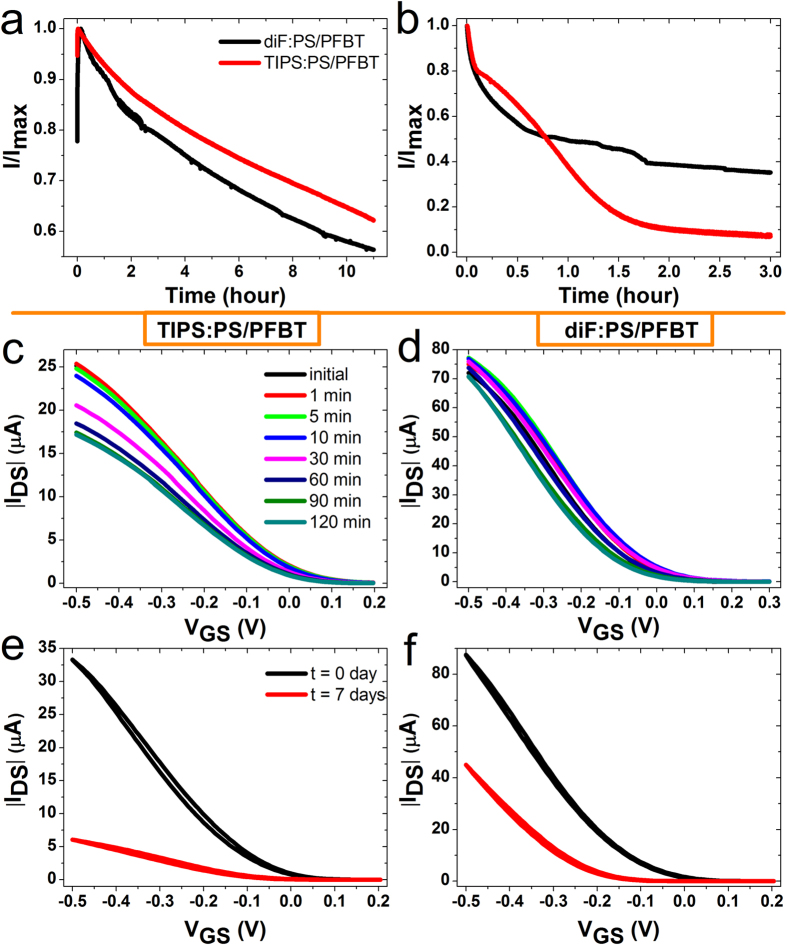
*I-t* plot of TIPS:PS/PFBT- (red curves) and diF:PS/PFBT-based (black curves) devices operated in (**a**) MilliQ water and (**b**) NaCl 1M. Both measurements were carried out in saturation regime (*V*_*DS*_ = −0.4 V, *V*_*GS*_ = −0.4 V). Overlay of *I-V* transfer characteristics for the bias-stress experiment for (**c**) TIPS:PS/PFBT and (**d**) diF:PS/PFBT-based devices up to two hours. For sake of clarity, only some measurements have been shown. All the *I-V* transfer characteristics have been automatically recorded after 1 minute of electrical stress (*V*_*GS*_ = −0.4 V, *V*_*DS*_ = −0.4 V). All the experiments have been performed in MilliQ water. Overlay of the *I-V* transfer characteristics at the beginning (black line) and after one week (red line) for (**e**) TIPS:PS/PFBT and (**f**) diF:PS/PFBT-based devices.

As stated in the introduction, the electrical stability is an open issue in the field of organic electronics operated in aqueous media^15^,^16^. The majority of EGOFETs devices provide reliable data for standard experiments demanding only single-spot measurements within a timescale of a few hours. This limited water stability restricts the number of organic semiconductors that can be applied in EGOFETs and is surely the main bottleneck that hampers the real application of these devices. According to our electrical characterization, no signs of electrochemical doping have been observed^17^. For all these reasons, we undertook a manifold approach in order to investigate in depth the stability of the fabricated EGOFETs. *In-situ* real-time monitoring, bias stress and shelf stability are the three different ways of testing. The first one consists of a continuous device measurement in saturation regime (*i.e. V*_*GS*_ = −0.4 V and *V*_*DS*_ = −0.4 V). In MilliQ water (Fig. 4a), TIPS:PS/PFBT and diF:PS/PFBT show a similar behavior, where *I*_*DS*_ undergoes a first transient increase (namely, within 30 min) and a consequent slow decrease. The total *I*_*DS*_ loss is around 35 ÷ 40% within 11 hours. In NaCl 1M (Fig. 4b), the two active materials differ in *I*_*DS*_ lowering, being the effective loss 31%/hour and 25%/hour for TIPS:PS/PFBT and diF:PS/PFBT, respectively (see Table S2). This clearly shows that diF-TES-ADT is more stable than TIPS-pentacene, even though both EGOFETs are still effectively working with weaker performances. This difference in stability between both materials could be attributed to a more favorable OSC/electrolyte interface in diF-based devices probably due the larger crystallites present in these thin films. In addition, the increased ionic strength of the aqueous media does not allow us to observe the transient *I*_*DS*_ increase, which is present when we were using MilliQ water. This is likely due to the slower polarization process in MilliQ water compared to saline solutions. It should be emphasized that despite the devices are significantly diminishing their performance upon continuous operation, to our knowledge, there are no precedents of EGOFETs working for such a long time in these conditions^15^. Bias stress measurements were also carried out, as shown in Fig. 4c,d and Fig. S12a,b. These measurements consist of an automatic *I-V* transfer recording after 1 minute of continuous stress (see Methods)^38^. This is a common device characterization test in standard OFETs devices, but has not been carried out before in water gated transistors. The results are completely consistent with the previous ones. It has been not only observed the electrical polarization occurring at the beginning of the experiment, but also the higher stability of diF:PS/PFBT with respect to TIPS:PS/PFBT. As a result, the former shows a small *V*_*ON*_ shift during 2 hours of stress, whereas the latter undergoes a worsening in mobility after 30 minutes of bias stress. Besides real-time monitoring and bias stress tests, another type of electronic application might need few single-spot measurements within a long period of time. Aiming at this particular usage, the so-called shelf-stability has been devised (Fig. 4e,f), in which *I-V* transfer characteristics have been recorded once per day up to one week. These experiments consist of measuring, drying and storing the device each day. Again, diF:PS/PFBT-based EGOFET is mostly affected by a negative *V*_*ON*_ shift, whereas the TIPS:PS/PFBT device is additionally affected by a mobility worsening. However, both devices still work within this period, even in the harsher aqueous media (see Fig. S12c,d). All these tests not only prove the good electrical performances of the devices but also point towards their potential applicability in saline aqueous media. The superior operational stability of EGOFET in aqueous solutions even at high ionic strength represents a major breakthrough for potential applications for long-term monitoring. These features are ascribed to the high degree of crystallinity of these films. This is achieved thanks to the combination of the BAMS technology with the blending of the organic semiconductors with polystyrene, which promotes processability and an enhanced crystallization of the semiconductor. In fact, the lack of holes and/or inhomogeneities in the films confer excellent features for the realization of robust EGOFETs.

## Conclusions

In summary, we have systematically studied the electrical performances of two soluble OSCs, namely diF-TES-ADT and TIPS-pentacene blended with polystyrene. Although these two materials have been extensively used up to date in organic electronics, this is the first report where they have been exploited in EGOFETs. We succeeded in using bar-assisted meniscus shearing, whose capability to produce homogenous and crystalline thin films at long range length scale has been pivotal. In addition, the cooperative effect of both PFBT and PS has been crucial to optimize the thin film morphology. This work does not only widen the library of OSCs suitable for EGOFETs, but also leads to devices extremely efficient and robust with unprecedented performance. Even though the differences between the two active materials are few, diF:PS/PFBT appears to be the best option in terms of long-term measurements, although TIPS:PS/PFBT is more sensitive to potentiometric changes. In conclusion, the combination of blended materials along with BAMS has been clearly demonstrated to be a powerful route for EGOFETs manufacturing in order to solve their open challenges.

## Methods

### Materials

6,13-Bis(triisopropylsilylethynyl)pentacene (TIPS-pentacene), 2,8-difluoro-5,11-bis(triethylsilylethynyl)anthradithiophene (diF-TES-ADT) were purchased from Ossila and Lumtec., respectively. Polystyrene (M_w_ ~ 10,000 g mol^−1^), Chlorobenzene (CB) and 2,3,4,5,6-Pentafluorothiophenol (PFBT) were purchased from Sigma-Aldrich and used as received. Polydimethilsiloxane (PDMS) was purchased from Dow Corning Corporation and prepared according to the standard protocol. Platinum (Pt) wire (ø = 0.5 mm) was purchased from Sigma-Aldrich and cleaned with sulphuric acid (0.1M) and MilliQ water (resistivity: at 25 °C 18.2 MΩ. cm)d prior to be used as a top gate electrode.

### Device fabrication

A heavily doped n-type silicon wafer featuring thermal SiO_x_ (200 nm thick) was used as substrate for our devices; source and drain (S/D) electrodes were defined by photolithography (MicroWriter ML^TM^ Laser Lithography System) and a metallic layer of Cr/Au (5 nm/40 nm) was subsequently evaporated (BOC Edwards Auto 360). The last step of the electrodes fabrication is the so called “lift-off” that consists in the immersion of our substrates in acetone. The channel width (*W*) and length (*L*) were 20700 μm and 30 μm (namely having a geometrical ratio *W*/*L* = 690), respectively. Prior to the deposition of the soluble OSC by using BAMS, the substrates were cleaned in ultrasonic bath with acetone and isopropanol for 15 min respectively and afterward ozone-treated for 25 min. S/D electrodes were subsequently modified by immersing the device for 15 minutes in a 15 mM PFBT solution in isopropanol. After PFBT functionalization, the excess of SAM was removed by a further ultra-sonication cleaning step in fresh isopropanol for 5 minutes. The devices lacking of the PFBT functionalization have been further immersed in a simple isopropanol solution for 15 min. The OSC and polymer were mixed in a 4:1 ratio, and then dissolved in chlorobenzene reaching a final concentration of 2 wt%. The blend solution was kept on a hot-plate at 105 °C for 1 h to ensure the complete dissolution of the two components. The blends coating were realized in ambient conditions through a home-adapted bar coater working at fixed speed of 1 cm s^−1^ and keeping the substrate temperature at 105 °C.

### Electrical measurements

All electrical characteristics were performed under ambient conditions using an Agilent 5100A and Easy Expert software connected to the samples with a SÜSS probe station. The transfer characteristics were recorded at a scan rate of 60 mV/s. A Pt wire acts as top gate electrode and the liquid electrolyte was confined on the interdigitated region by means of a PDMS pool (total volume was equal to 20 μL); for all the stability measurements a close PDMS reservoir (200 μL) was employed in order to minimize solvent evaporation throughout the electrical test. For the *I-t* monitoring, *V*_*GS*_ and *V*_*DS*_ are fixed to −0.4 V and *I*_*DS*_ were continuously recorded. Regarding the bias stress measurements, a transfer characteristics and a stressing cycle (*V*_*GS*_ = −0.4 V, *V*_*DS*_ = −0.4 V, t = 1 minute) are alternatively applied. Displacement current measurements (DCM) and potentiometric sensitivity test were performed with the same equipment used for the standard electrical characterization. For the DCM measurement, the source and drain electrodes were short-circuited and grounded. The scan rates of the gate voltage were changed and the gate currents were recorded. For the potentiometric sensitivity measurements, a square pulse (*ΔV*_*GS*_) of different amplitudes (from 50 mV to 100 μV) was applied with respect to the starting *V*_*GS*_ equal to −0.4 V with an integration time of 20 ms. Switching speed measurements were recorded by using Keithley 2604B SMU controlled with a software provide by the company. Here, the *I*_*DS*_ current is recorded when *V*_*GS*_ is swept from 0 V to −0.5 V (with an integration time of ~270 μs). For electrochemical impedance spectroscopy measurement (EIS), the same S/D electrodes configuration, as used for DCM measurements, were employed. EIS response was monitored by a Novocontrol Alpha-AN impedance analyzer equipped with POT/GAL 30 V/2A electrochemical interface in a frequency range of 10^6^ ÷ 10^−1^ Hz. DCM and EIS measurements were scaled according to a transistor area of 0.0208 cm^2^.

### Polarizer Optical microscopy and AFM image

Optical microscope images were obtained using an Olympus BX51 equipped with polarizer and analyzer. Surface topography was examined by a 5500LS SPM system from Agilent Technologies and subsequent data analysis was performed by using Gwyddion 2.41 software.

## Additional Information

**How to cite this article**: Zhang, Q. *et al*. High performing solution-coated electrolyte-gated organic field-effect transistors for aqueous media operation. *Sci. Rep.* **6**, 39623; doi: 10.1038/srep39623 (2016).

**Publisher's note:** Springer Nature remains neutral with regard to jurisdictional claims in published maps and institutional affiliations.

## Supplementary Material

Supplementary Information

## Figures and Tables

**Table 1:** Field effect mobility (*μ*), threshold voltage (*V*_*th*_), on/off ratio (*I*_*on*_/*I*_*off*_), and subthreshold swing (*SS*) of our EGOFETs are extracted in saturation regime.

system	MilliQ H_2_O				NaCl 1M			
	*μ* (cm^2^V^−1^s^−1^)*	*V*_*th*_ (mV)	*I*_*on*_/*I*_*off*_	*SS*(mV/dec)	*μ* (cm^2^V^−1^s^−1^)*	*V*_*th*_ (mV)	*I*_*on*_/*I*_*off*_	*SS* (mV/dec)
TIPS:PS/PFBT	0.12	12 (±2)	7.3 × 10^3^	87 (±3)	0.07	−166 (±5)	1.0 × 10^4^	77 (±6)
TIPS:PS	0.05	−112 (±3)	6.1 × 10^3^	80 (±2)	0.02	−194 (±5)	3.7 × 10^3^	87 (±9)
TIPS/PFBT	0.002	−79 (±1)	1.1 × 10^2^	184(±1)	0.001	−156 (±3)	1.8 × 10^2^	100 (±5)
TIPS	0.001	−51 (±1)	8.3 × 10^1^	211(±7)	0.001	−129 (±1)	4.1 × 10^1^	208 (±7)
diF:PS/PFBT	0.18	49 (±1)	1.5 × 10^4^	82 (±2)	0.08	−19 (±1)	9.7 × 10^3^	86 (±7)
diF:PS	0.10	125 (±1)	4.3 × 10^3^	87 (±7)	0.07	−152 (±4)	8.6 × 10^3^	86 (±7)
diF/PFBT	0.04	−145 (±3)	1.2 × 10^3^	97 (±5)	0.02	−200 (±4)	1.6 × 10^3^	87 (±5)
diF	0.004	−28 (±4)	1.6 × 10^2^	121(±1)	0.02	−120 (±2)	5.5 × 10^2^	90 (±4)

^*^All these values are affected by an error ranging from 2% to 20%.

**Table 2:** Values of switch on (*τ*_*on*_) and switch off (*τ*_*off*_) speeds in MilliQ H_2_O.

System	*τon* (ms)	*τ*_*off*_ (ms)
TIPS:PS/PFBT	0.45 (±0.04)	0.25 (±0.02)
diF:PS/PFBT	0.65 (±0.07)	0.31 (±0.03)
