# Supplementary material for: High performing solution-coated electrolyte-gated organic field-effect transistors for aqueous media operation
Source: Sci Rep. 2016 Dec 22;6:39623. doi: 10.1038/srep39623 (PMC5177926; doi:10.1038/srep39623)
Supplement: Supplementary Information [file srep39623-s1.pdf]

Supporting Information

High performing solution-coated electrolyte-gated organic field-effect transistors for aqueous media operation

*Qiaoming Zhang, Francesca Leonardi, Stefano Casalini, Inés Temiño and Marta Mas-Torrent**

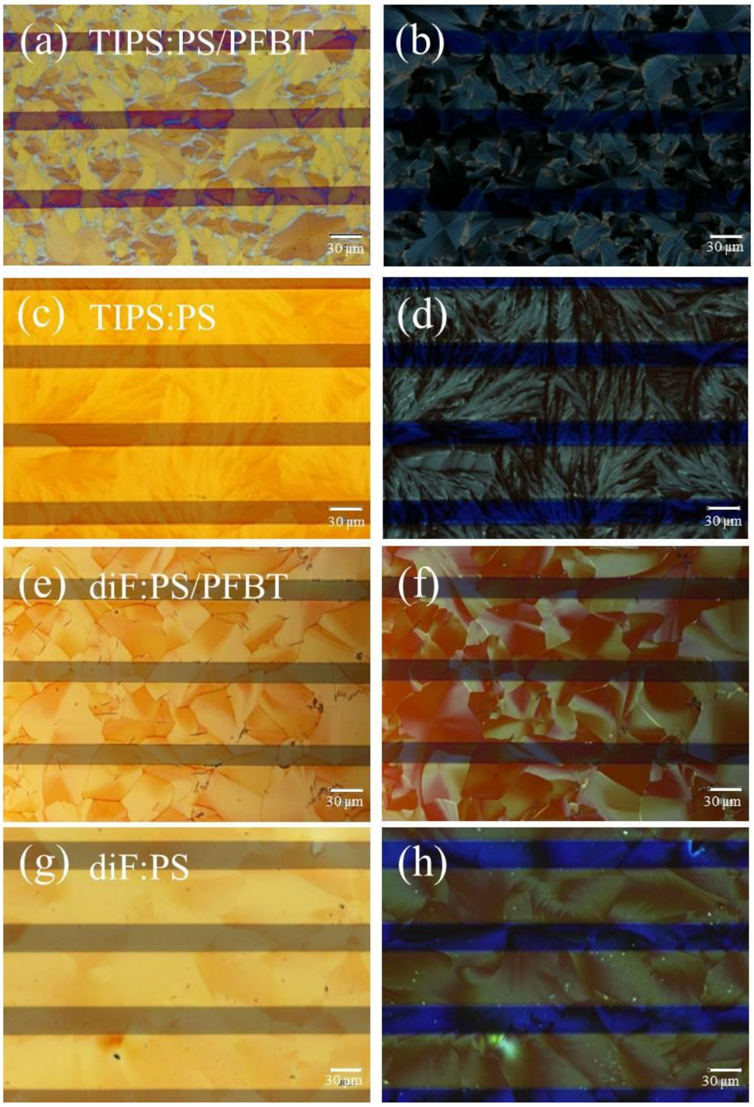

Figure S1 The optical microscopy images acquired with a cross polarizer. On the left, polarizer/analyzer = 0°. On the right, polarizer/analyzer = 90°.

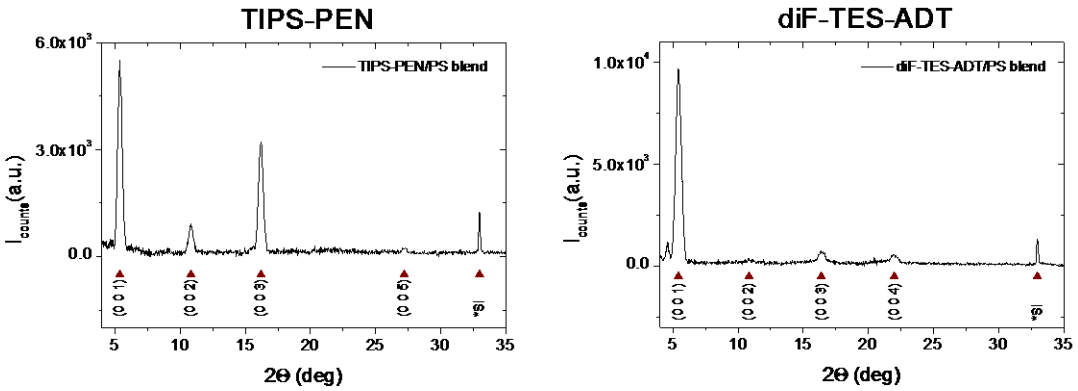

Figure S2 X ray diffractograms of thin films based on TIPS-PEN and diF-TES-ADT. The peak placed approximately at 33° is due to the silicon substrate reflection.

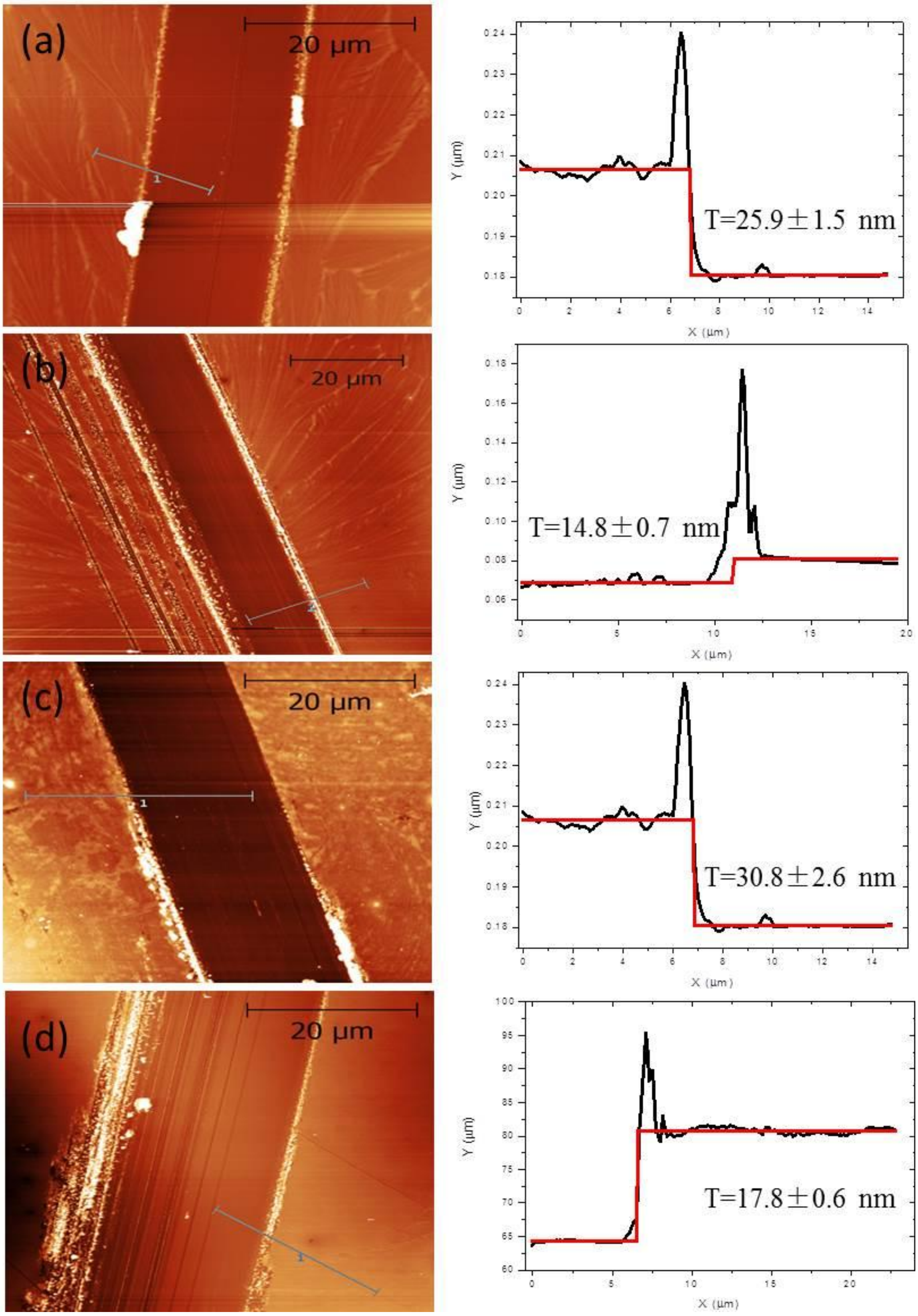

Figure S3 AFM profiles and their corresponding 2D fitting of a) TIPS:PS/PFBT, b) TIPS:PS, c) diF:PS/PFBT and d) diF:PS devices.

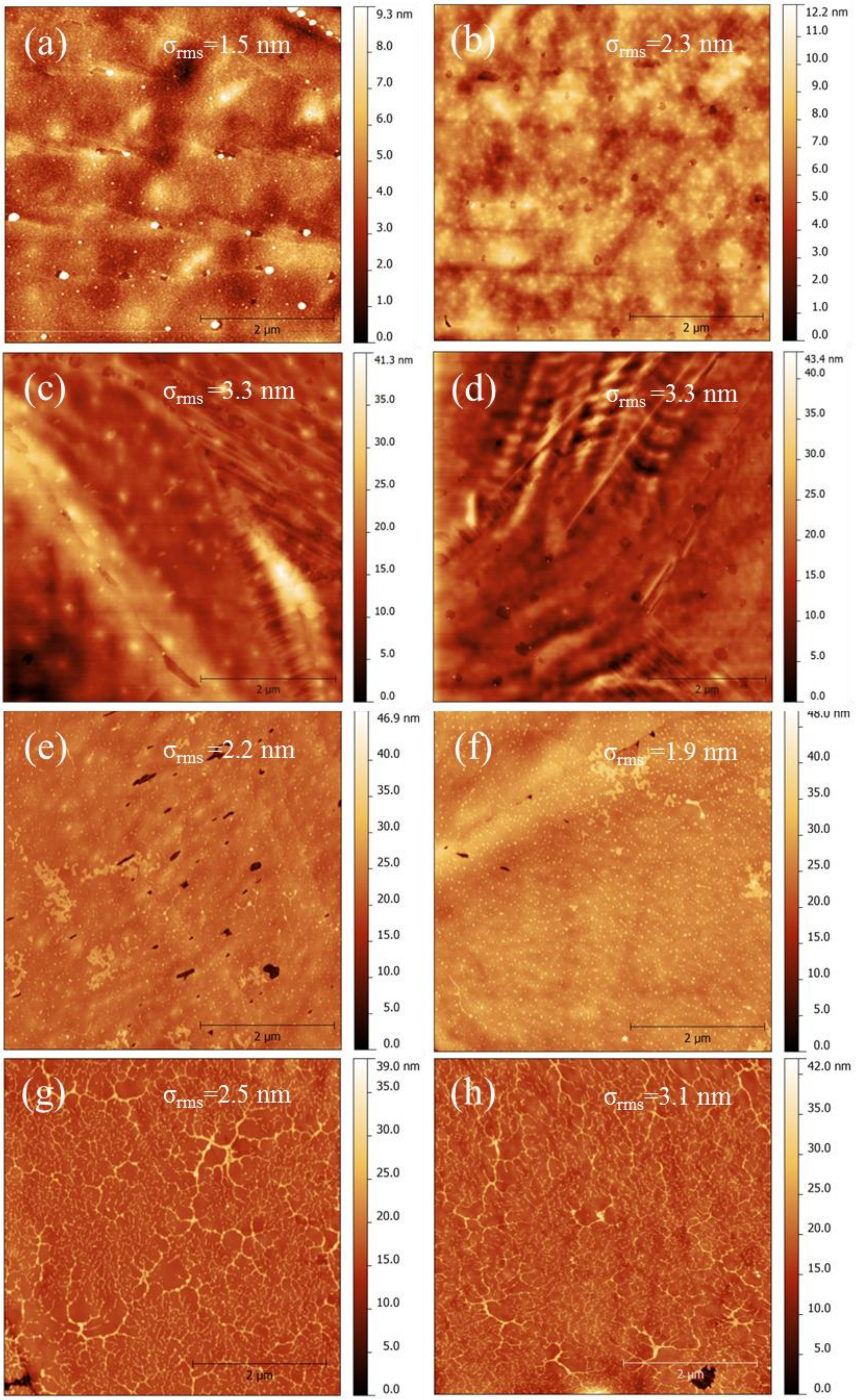

Figure S4 AFM images (5×5 µm^2) of a) b)TIPS:PS/PFBT, c) d) TIPS:PS, e) f) diF:PS/PFBT and g) h) diF:PS devices. The left column images were acquired on the channel region, while the ones on the right on the electrodes region.

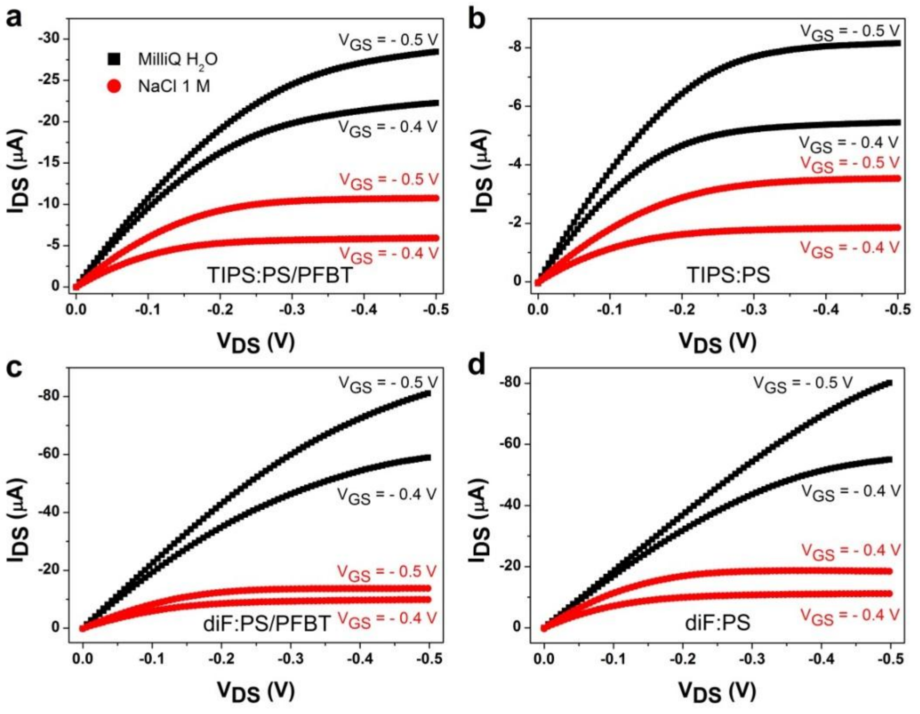

Figure S5 Output characteristics of a) TIPS:PS/PFBT, b) TIPS:PS, c) diF:PS/PFBT and d) diF:PS recorded in MilliQ H_2O (black curves) and NaCl 1M (red curves)

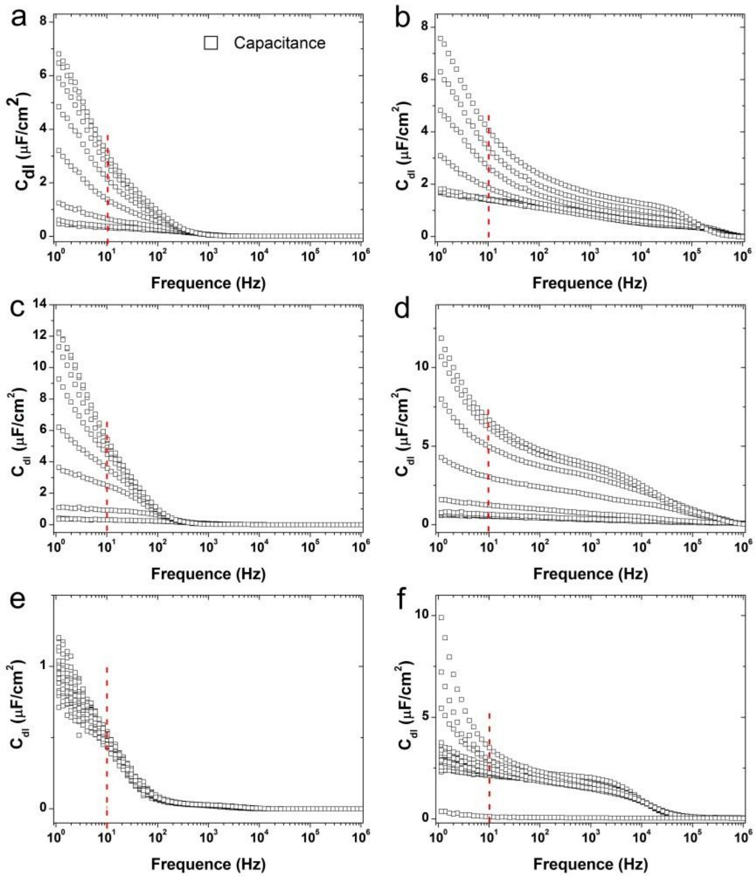

Figure S6 EIS measurements of TIPS:PS/PFBT device recorded at different DC voltages (from 0.1V to -0.5 V with a pace of -0.1V) under a) MilliQ H_2O and b) NaCl 1 M. EIS measurements of diF:PS/PFBT device in c) MilliQ H_2O and d) NaCl 1 M. EIS measurements of the uncoated device in e) MilliQ H_2O and f) NaCl 1 M.

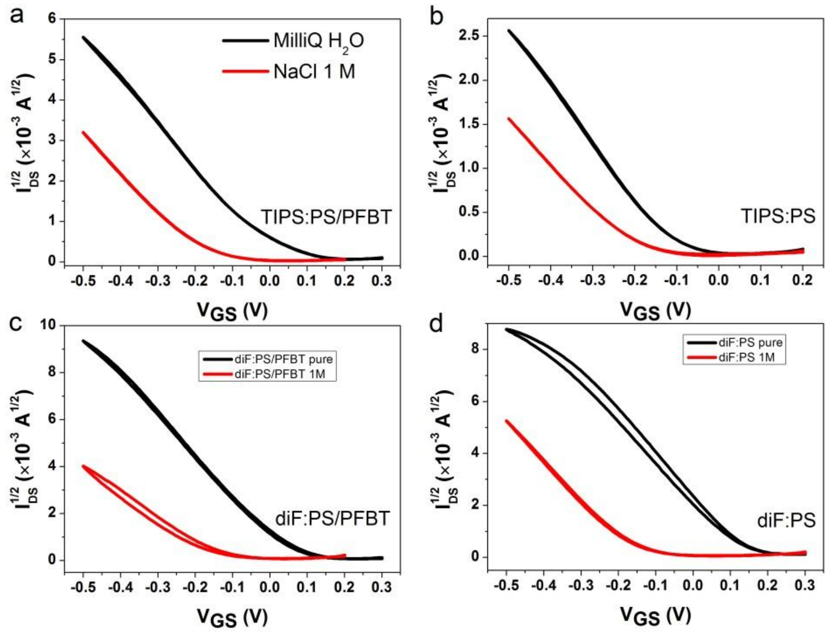

Figure S7 I-V transfer characteristics of a) TIPS:PS/PFBT, b) TIPS:PS, c) diF:PS/PFBT, d) diF:PS recorded in MilliQ H_2O and NaCl 1M.

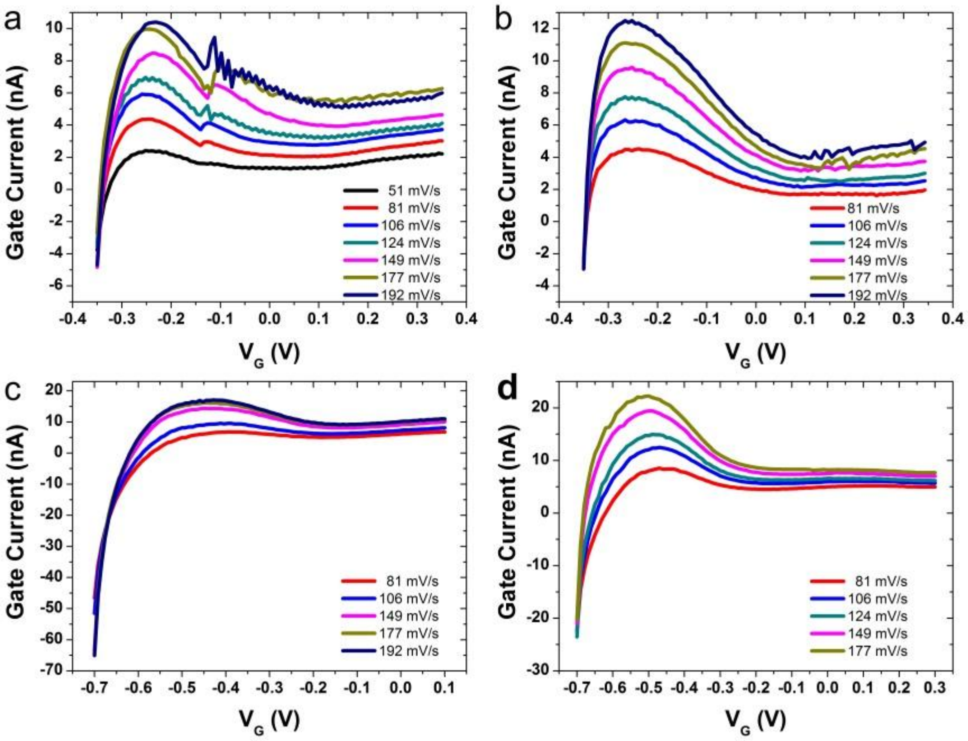

Figure S8 Overlay of displacement current measurements at different scan rates of TIPS:PS/PFBT in a) MilliQ H_2O and b) NaCl 1 M. c) and d) The same results of DCM for diF:PS/PFBT devices under MilliQ H_2O and NaCl 1 M.

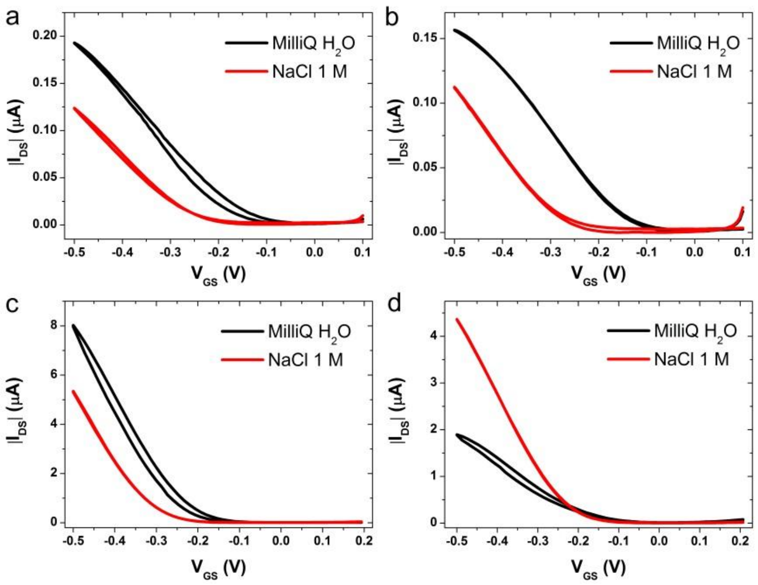

Figure S9 I-V transfer characteristics (V_{DS}= -0.4 V) of a) TIPS/PFBT, b) TIPS, c) diF/PFBT and d) diF devices.

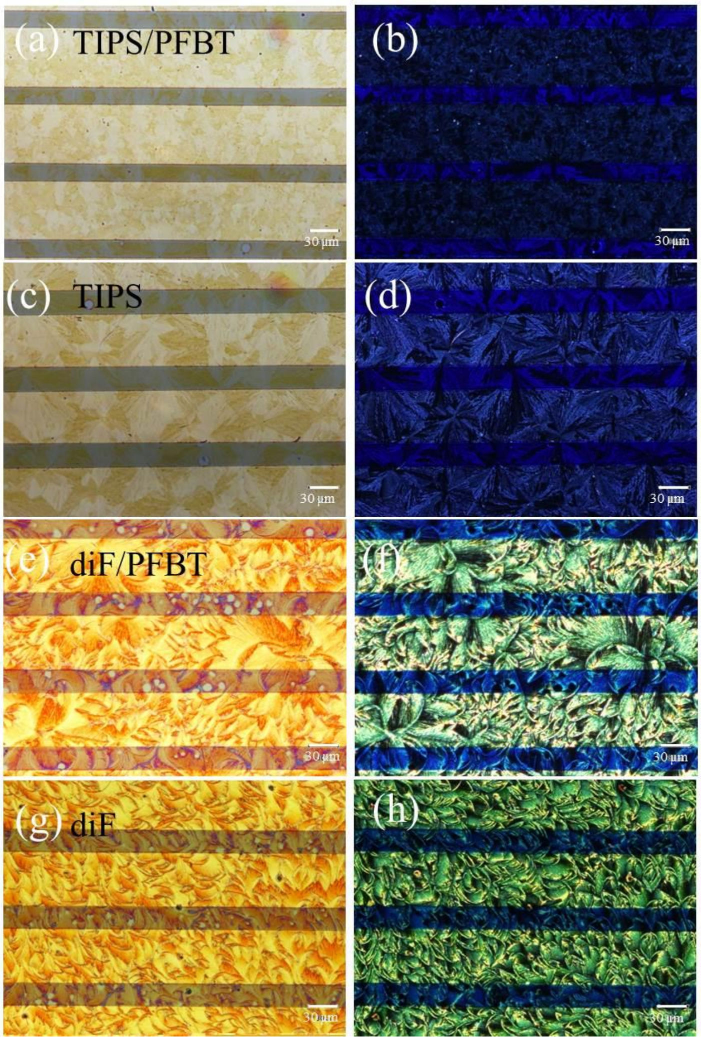

Figure S10 Optical microscopy images acquired with a cross polarizer. On the left, polarizer/analyzer = 0°. On the right, polarizer/analyzer = 90°.

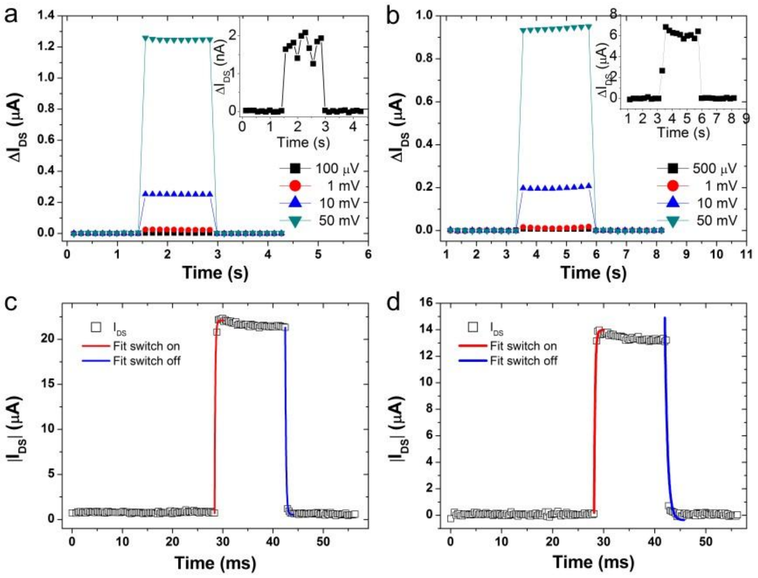

Figure S11 Potentiometric sensitivity of a) TIPS:PS/PFBT and b) diF:PS/PFBT devices. The different V_{GS} steps are listed in the corresponding graph. Switching speed response of c) TIPS:PS/PFBT and d) diF:PS/PFBT devices. All the measurements are recorded in NaCl 1M at V_{DS} = - 0.4 V.

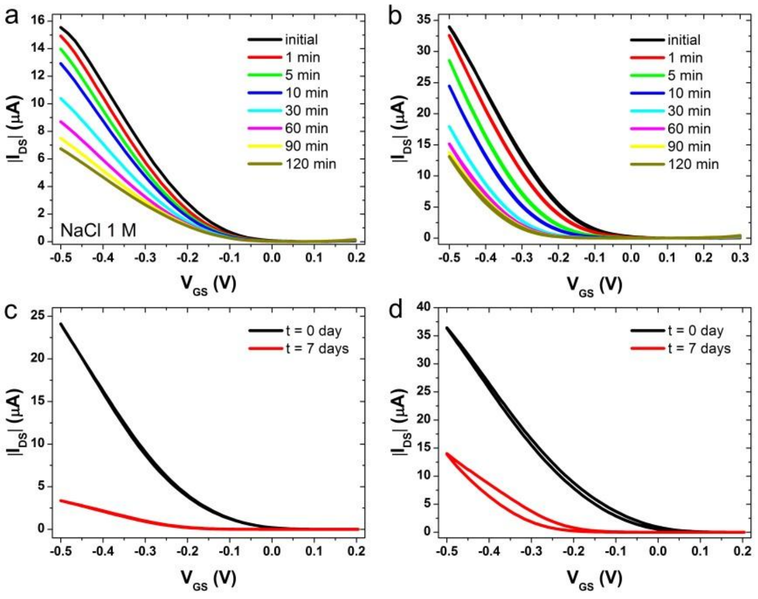

Figure S12 Bias stress experiment of a) TIPS:PS/PFBT and b) diF:PS/PFBT devices. Shelf-stability of our EGOFETs recorded at first and seventh day: c) TIPS:PS/PFBT and d) diF:PS/PFBT devices. All the measurements were performed in NaCl 1 M in saturation regime (V_{DS} = -0.4 V).

Table S1 Values of capacitance extracted by DCM and EIS.

	Capacitance (μF/cm^2) (Method: DCM)		Capacitance (μF/cm^2) (Method: EIS)	
	MillQ H_2O	NaCl 1 M	MillQ H_2O	NaCl 1 M
TIPS:PS/PFBT	2.6	3.4	2.9	3.3
diF:PS/PFBT	4.5	7	5.3	6.1

Table S2 The degradation speeds for *in-situ* current monitoring and shelf-stability in MilliQ H_2O and NaCl 1 M solution.

	Current monitoring (%/hour)		Shelf stability(%/day)	
	MilliQ H_2O	NaCl 1 M	MilliQ H_2O	NaCl 1 M
TIPS:PS/PFBT	3.4	30.9	11.6	12.3
diF:PS/PFBT	3.9	21.6	6.9	9.1
